# An Electronic Data Capture Tool for Data Collection During Public Health Emergencies: Development and Usability Study

**DOI:** 10.2196/35032

**Published:** 2022-06-09

**Authors:** Joan Brown, Manas Bhatnagar, Hugh Gordon, Jared Goodner, J Perren Cobb, Karen Lutrick

**Affiliations:** 1 Clinical Operations Business Intelligence The Keck School of Medicine of the University of Southern California Los Angeles, CA United States; 2 Department of Surgery The Keck School of Medicine of the University of Southern California Los Angeles, CA United States; 3 Akido Labs Inc Los Angeles, CA United States; 4 Department of Family and Community Medicine University of Arizona Tucson, AZ United States

**Keywords:** clinical research design, disaster management, informatics, public health emergencies, electronic data capture, design tenet, public health emergency, electronic data, EDCT, real time data

## Abstract

**Background:**

The Discovery Critical Care Research Network Program for Resilience and Emergency Preparedness (Discovery PREP) partnered with a third-party technology vendor to design and implement an electronic data capture tool that addressed multisite data collection challenges during public health emergencies (PHE) in the United States. The basis of the work was to design an electronic data capture tool and to prospectively gather data on usability from bedside clinicians during national health system stress queries and influenza observational studies.

**Objective:**

The aim of this paper is to describe the lessons learned in the design and implementation of a novel electronic data capture tool with the goal of significantly increasing the nation’s capability to manage real-time data collection and analysis during PHE.

**Methods:**

A multiyear and multiphase design approach was taken to create an electronic data capture tool, which was used to pilot rapid data capture during a simulated PHE. Following the pilot, the study team retrospectively assessed the feasibility of automating the data captured by the electronic data capture tool directly from the electronic health record. In addition to user feedback during semistructured interviews, the System Usability Scale (SUS) questionnaire was used as a basis to evaluate the usability and performance of the electronic data capture tool.

**Results:**

Participants included Discovery PREP physicians, their local administrators, and data collectors from tertiary-level academic medical centers at 5 different institutions. User feedback indicated that the designed system had an intuitive user interface and could be used to automate study communication tasks making for more efficient management of multisite studies. SUS questionnaire results classified the system as highly usable (SUS score 82.5/100). Automation of 17 (61%) of the 28 variables in the influenza observational study was deemed feasible during the exploration of automated versus manual data abstraction. The creation and use of the Project Meridian electronic data capture tool identified 6 key design requirements for multisite data collection, including the need for the following: (1) scalability irrespective of the type of participant; (2) a common data set across sites; (3) automated back end administrative capability (eg, reminders and a self-service status board); (4) multimedia communication pathways (eg, email and SMS text messaging); (5) interoperability and integration with local site information technology infrastructure; and (6) natural language processing to extract nondiscrete data elements.

**Conclusions:**

The use of the electronic data capture tool in multiple multisite Discovery PREP clinical studies proved the feasibility of using the novel, cloud-based platform in practice. The lessons learned from this effort can be used to inform the improvement of ongoing global multisite data collection efforts during the COVID-19 pandemic and transform current manual data abstraction approaches into reliable, real time, and automated information exchange. Future research is needed to expand the ability to perform automated multisite data extraction during a PHE and beyond.

## Introduction

Knowledge sharing during public health emergencies (PHE) is critical to managing swift and appropriate responses by key decision makers. Moreover, clinical responsibilities are typically increased, and dedicated research personnel may be lacking during a PHE. Despite the call to action from the medical community placed on data sharing for effective response, there remains a lack of standard best practice on information exchange during PHE, with no widely available platform mechanism to facilitate data sharing [1-4]. The absence of standards and technology challenges the ability of clinicians to develop a unified treatment plan to confront patients exposed to the PHE at hand. This has been evident since 2001, when the US Public Health System was challenged with the threat of an Anthrax outbreak [5]. Disparate information sources and unclear jurisdiction across local, state, and federal agencies prevent accurate knowledge sharing and aligned recommendations from decision makers [5]. The lack of information during PHE is a global challenge, as demonstrated in the data collection efforts during the Zika virus epidemic, Ebola outbreak [6], and most recently the COVID-19 pandemic [7-9]. This has been exacerbated during the COVID-19 pandemic, where data are needed to guide treatment protocols, but data sharing across a global spectrum is nonexistent or delayed [9-12]. Global standards and a system that allows for real-time learning during public health crisis are critical to our health care community’s ability to respond to PHE [7-9,13-18].

Optimal responses to PHE require data-driven approaches that allow for prospective and real-time clinical data collection and dissemination that overcome the various challenges in data quality [18]. The current systems suffer from inadequate infrastructure for multisite clinical data capture [8,16,19,20], delays in dissemination of data due to lack of technical capacity [21], a lack of tools to manage the quality of data [20], and the absence of simple and straightforward interfaces that do not add to clinical burden of data collection during PHE [18]. To mitigate the known barriers to data collection during PHE, the Discovery Critical Care Research Network Program for Resilience and Emergency Preparedness (Discovery PREP [17]) partnered with Akido Labs, a third-party technology vendor, to develop a platform known as Project Meridian, a tool designed for data capture and dissemination during PHE. Discovery PREP’s experience with current research data capture platforms during national health system stress tests, and other PHE tabletop exercises, indicated excessive person-hour effort required to coordinate data collection from multiple sites in a simulated PHE [22-25]. Thus, Discovery PREP began investigating novel methods toward multisite clinical data extraction with the goal of significantly increasing the nation’s capability to manage real-time clinical data collection and analysis during PHE. Exploration proceeded with the design and development of a technology-agnostic electronic data capture tool that could facilitate multisite automated data extraction and storage. Following the development of the electronic data capture tool, the feasibility of advancing data capture using automated data extraction compared to manual data entry was assessed in 2 observational studies [26-29]. This paper describes the technical process and lessons learned from this effort, concluding with recommendations for improvement of data sharing platforms during PHE.

## Methods

### Overview

**Figure 1:**
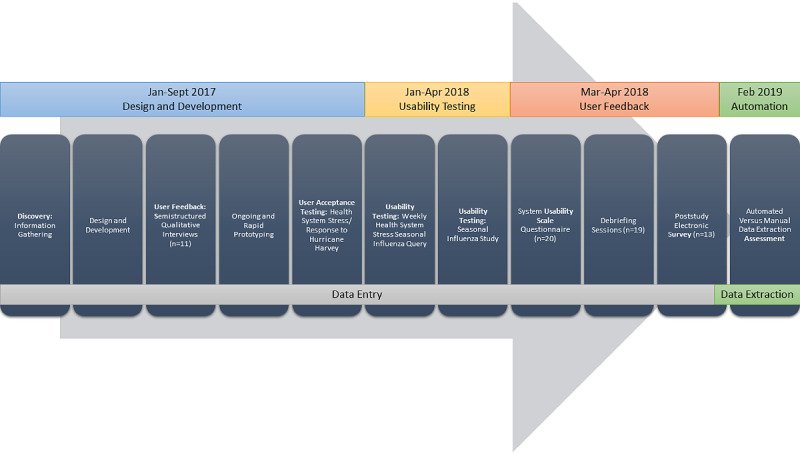
Timeline.

A multiyear and multiphase approach was taken to develop the electronic data capture tool as visualized in the design timeline (Figure 1). The tool was first developed and piloted for rapid data capture and then expanded to assess the feasibility of automated clinical data extraction. The design and evaluation of the electronic data capture tool spanned from January 2017 to April 2018.

Reducing the burden of data collection was a key design principle for the electronic data capture tool, as clinical responsibilities typically increase during PHE, and the availability of research personnel is insufficient to capture the volume of data need for robust clinical trials and their analysis, especially for the critically ill or injured [2,3,18]. The electronic data capture tool was designed with an intuitive data entry interface to reduce time and effort for data entry with the added capability to enter data on a smartphone. Ease of use was combined with considerations for scalability across multiple institutions to eliminate manual administration processes and bridge the gap created by disparate platforms.

### Platform Design and Development

The participants included Discovery PREP physicians, their local administrators, and data collectors from tertiary-level academic medical centers at 5 geographically distributed institutions (University of Southern California, Washington University in St. Louis, Baylor University, Mayo Clinic, and Duke University). Design, development, rapid prototyping, and user feedback took place between January 2017 and July 2017. Information was gathered prior to the development of the electronic data capture tool to identify the unmet needs and solidify design specifications. Individual semistructured interviews were conducted over a 45-minute duration. A total of 11 participants were interviewed, including 3 (27%) physician researchers, 3 (27%) data collectors, 4 (36%) administrators, and 1 (9%) biostatistician to understand workflows and data collection challenges during a PHE. The initial predevelopment interviews were qualitative in nature and elicited information on data collection processes and limitations of current electronic data capture tools (Table 1).

**Table 1:** Qualitative interview questions prior to electronic data capture tool development.

Question #	Question detail
1	When do you complete CRFs^a^ with respect to enrollment time?
2	How are new study subjects identified?
3	How are new subjects communicated to data collector?
4	What is your process for collecting data for the CRF?
5	What are the pain points you experience with REDCap?^b^
6	Pain points with the last study you participated in?

^a^CRF: case report form.

^b^REDCap: Research Electronic Data Capture.

**Figure 2:**
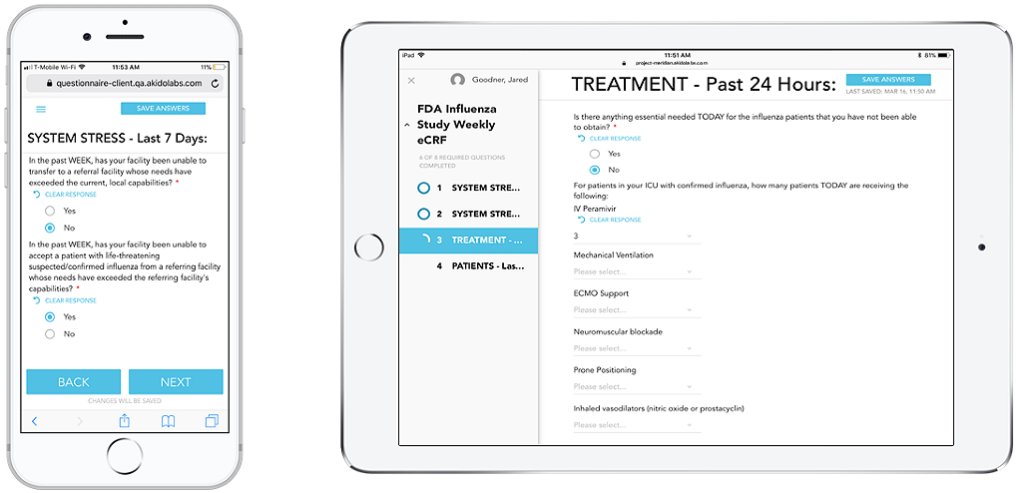
Project Meridian mobile capability screenshots. ECMO: extracorporeal membrane oxygenation; eCRF: Electronic Case Report Form; FDA: Food and Drug Administration; ICU: intensive care unit; IV: intravenous.

Project Meridian was designed to be powered by the Akido Labs Development Environment. The latter was designed to enable modern development in a health care environment by abstracting four core unique complexities specific to this industry, including security of patient health information, compliance, interoperability, and governance (Multimedia Appendix 1). User-centered design practices with an eye toward a simple user interface were the basis of the design of the user interface and prototypes (Figure 2; Multimedia Appendices 2 and 3). Postdevelopment, user prototyping interviews focused on feedback regarding electronic data capture tool prototypes. An agile approach of rapid iteration following user feedback was taken to enhance the electronic data capture tool following each interview in preparation for the next, following a hypothesize-design-test learning loop. Interviews were performed both in person and remotely via screen sharing, as needed. The interviews were semistructured, and users conducted standardized tasks while observed by the investigator team including the following: Discovery PREP administrative team, Akido Labs engineers, and a notetaker to capture user feedback on functionality, user experience, and messaging to guide usage. All clicks, mouse movements, and time required to accomplish specific tasks were recorded for analysis and used to refine the platform design.

### Usability and Pilot Study Testing

**Figure 3:**
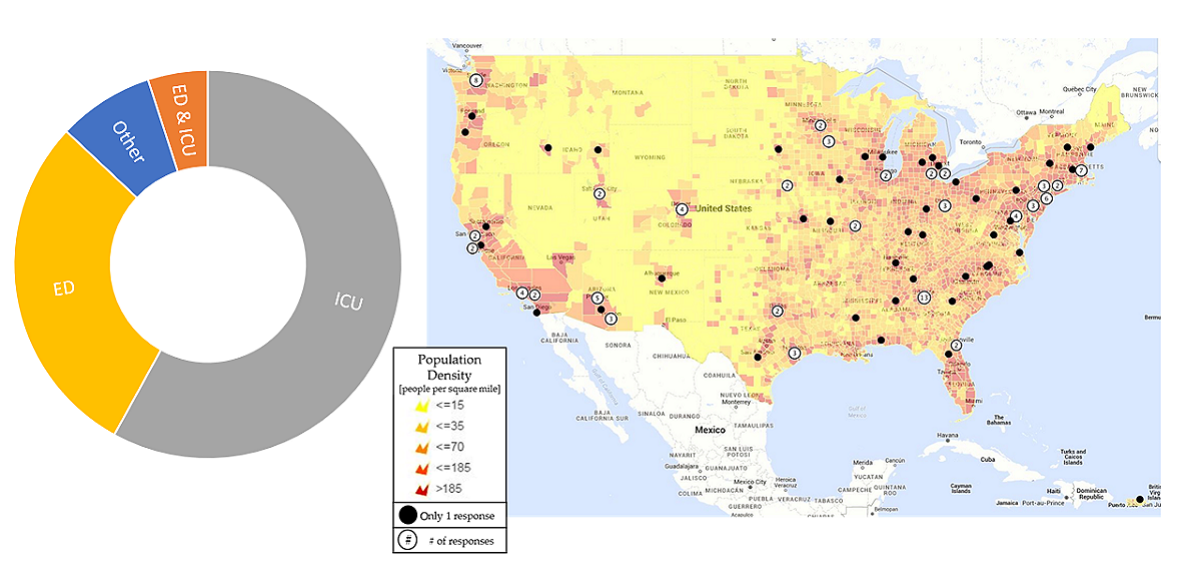
User acceptance testing (UAT) map of responders, National Health System Stress Query (n=401). ED: emergency department; ICU: intensive care unit.

**Figure 4:**
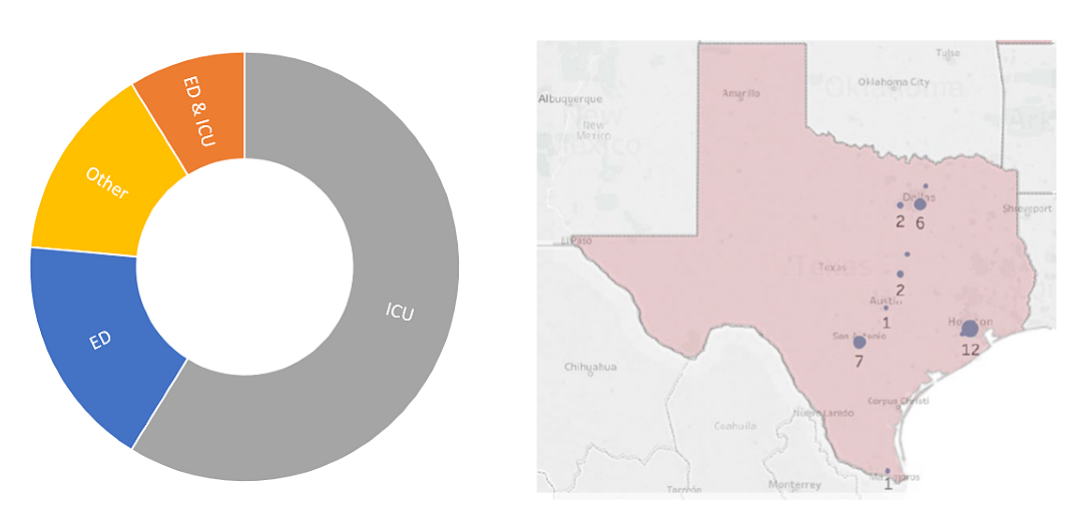
User acceptance testing (UAT) map of responders, Hurricane Harvey (n=20). ED: emergency department; ICU: intensive care unit.

Additional feedback was gathered during user acceptance testing (UAT). UAT was performed using the two following scenarios: (1) a Discovery PREP health system stress query over 401 participants in August 2017 (Figure 3), and (2) 34 Society of Critical Care Medicine participants affected by Hurricane Harvey in the state of Texas in September 2017 (Figure 4). The chart on the left for both Figures 3 and 4 show the breakdown of responder practice setting. The map in Figure 3 illustrates the map of responders superimposed on population density. The map in Figure 4 displays the number of responses to the Hurricane Harvey query. Additional feature enhancements were assessed based on the feedback gathered during UAT. Following UAT, the electronic data capture tool was used to facilitate data collection for 2 clinical studies encompassing 403 users across the United States. Both clinical studies involved gathering information on the impact of seasonal influenza on health system stress. The studies were conducted with 12 sites for 17 weeks. The first study involved a predefined set of users with a large data collection form including 151 patient-level clinical data elements. The second encompassed a brief data collection form with 20 questions with census and health system stress level data. Data were collected weekly from health care systems using the Project Meridian platform. Following these studies, a subset of users (n=20) completed the System Usability Scale (SUS) questionnaire [30,31], 19 (95%) participated in debriefing sessions, and 13 (65%) completed a poststudy survey.

### Ethics Approval

Both studies were approved under the University of Southern California Investigational Review Board (HS-16-00948).

### Automated Versus Manual Data Extraction

Seasonal influenza was used as a proxy for a PHE during the comparison of automated and manual data collection [29]. Patients in an intensive care unit (ICU) with laboratory-confirmed influenza were enrolled into an investigational review board–approved observational study (HS-17-00837). At a single institution, patient selection and data collection were completed using two methods in parallel: research personnel effort (manual) and querying of institutional clinical data warehouse (automated). Data were collected over a 2-week period using a consensus, previously reported tiered case report form (CRF). Tier 1 of the CRF sought demographics, diagnoses, and lab results as well as supportive care details from the first 24 hours of ICU visit. Tier 2 sought more detailed clinical data from disease onset to patient discharge. Tier 1 was used for comparison in the feasibility test. The automated approach required the identification of relevant patients and gathering of key data elements by executing daily automated queries to an institutional clinical data warehouse. Data were stored and compared for accuracy following the 2-week period.

## Results

### Platform Design and Development

During the design phase, the results of the initial qualitative interviews highlighted the following themes: (1) the need to automate data entry; (2) the need to automate frequent study communications and coordination tasks; (3) the importance of ease-of-access and usability; and (4) the need to enable real-time data reporting to stakeholders during a PHE. Identification of these needs led to the inclusion of multiple feature enhancements within the Project Meridian platform prior to product launch (Textbox 1).

**Project Meridian feature enhancements.**

**Feature description**

- Gamification—leaderboard for number of responses
- Text message–based survey initiation
- Refer a colleague (if primary responder not on clinical service, or new responder)
- Redesign of automated survey email (improving call to action)
- Improving visibility of case report form completion rate
- Common view for members of one study team (one institution). All case report forms visible to all study data collectors at a given institution
- Advanced query functions to prompt individuals or their sites

### Usability and Pilot Study Testing

Design, development, and UAT of the platform occurred over a 9-month period. During usability testing, using the observational studies, data entry personnel reported increased awareness of data entry completeness with the use of site level summary dashboards. Additionally, Discovery PREP study administrators reported that the automation of scheduled personalized emails to the study participants reduced study administration time by an estimated 80% compared to previous studies. The results of the SUS questionnaire [30,31] classified the system among the 90th percentile of a broad class of systems evaluated [30] and was therefore highly usable (SUS score 82.5/100).

### Automated Versus Manual Data Extraction

The automated and manual data extraction pilot for patient selection independently identified the correct patients (N=4) during the 2-week study period. Completion of Tier 1 of the CRF per patient was 100% (28/28) via manual approach and 61% (17/28) via automation. Compared with manually collected data, automated data were 50% (70/141) identical and 13% (18/141) different. Variables such as demographics, ventilator status, and availability of lab values were identical. The individual lab values pulled in the first 24 hours of ICU admission were not always identical as there were multiple values available for some patients within that first 24-hour period. Values for pregnancy status, preadmit events, coinfections, and means of identification were missing. Data obtained through automated means had an inherent delay of up to 24 hours due to the use of the data warehouse infrastructure. Manually collected data had an average delay of 2-days between fulfillment of inclusion criteria and enrollment into the study.

## Discussion

The electronic data capture tool designed and tested proved highly usable and capable of collecting critical information during PHE test scenarios. One of the lessons learned globally during the recent COVID-19 pandemic is the importance of standardized real-time data collection, analysis, and reporting [7,32]. Prior to the pandemic, Discovery PREP investigators and federal partners developed a novel data capture system to manage multisite data collection to address the all-hazards core data set used to characterize serious illness, injuries, and resource requirements during PHE [18]. The design and implementation of the Project Meridian electronic data capture tool was Discovery PREP’s successful solution to enhance coordinated data collection capabilities during PHEs by addressing the pain points experienced by the clinical community during multisite data collection. Discovery PREP continued to leverage and report on the use of the Project Meridian platform in subsequent national studies [33,34].

Throughout this design and use process, Discovery PREP learned that specific design tenets need to be addressed to successfully gather essential information during a PHE. These tenets include the following:

1. Gathering data to assess a nationwide health system stress during influenza seasons involved collecting data from a bedside clinician (N=403) or an individual institution (N=12) [26-28]. Thus, the data collection system needs to be scalable and adaptable to the number and type of participants.
2. The data gathered during an event may include multiple types of case report forms with a combination of similar and differing variables that often require repeat measurements. For example, one of the observational studies was a weekly query to assess health system stress, while the other study was a single-report event with the same set of variables but with additional clinical content. Furthermore, a common data dictionary was created across Discovery PREP participating institutions to ensure the alignment of data collection across the sites. Thus, a data collection system must be able to accommodate a common consensus data set, with repeated measures across studies, and aggregate data for analysis and reporting to regional and federal government agencies.
3. Automating study administrative and communication tasks (eg, reminder emails) reduced the amount of manual administration for the study. Additionally, the status board (eg, leaderboard) served as a self-service visual to assess individual responses compared to others and to drive an increase in participant response. Thus, a data collection system should automate communication tasks and incorporate a status board for self-service and to encourage participation, especially during PHEs such as the COVID-19 pandemic.
4. The participants noted that during a busy clinical shift, text messaging was a more effective way to obtain a rapid response. Thus, a data collection system needs to adapt to the preferred communication method of the participant, which may vary across time and institutions.
5. Automation of data and reduction of data acquisition time requires a highly interoperable system that integrates with the variety of platforms used at various institutions. Thus, a data collection system should provide the flexibility and functionality to integrate with local information technology infrastructure for automated and near–real time data capture.
6. In a single institution, the identification of eligible patients was reliably accomplished using automation. Additionally, 50% of the data collected manually for one of the observational studies was identically gathered through automation. However, when comparing the manual versus automated data extraction process, only discrete, categorical data fields were available. Text blocks within progress, operative, and discharge notes or the history and physical notes could not be automated for our purposes. Thus, an optimal data collection system should include natural language processing capability with access to these types of domains to fully automate local data extraction.

Extensive work is needed to meet the needs of rapid data collection during a PHE. This has been evident during the COVID-19 pandemic, where surveillance efforts have underlined the benefits of creating a Clinical Informatics Digital Hub for monitoring and for clinical trial data management [32]. To expand the findings in this report, more investigation is needed to assess the following: feasibility of real-time automation; the use of synchronization protocols as needed in areas challenged by unreliable or slow internet access [35]; the use of natural language processing to capture unstructured data [36]; and application of artificial intelligence to expand our ability to respond to a rapidly evolving disease [37]. With a lack of common regional or federal PHE reporting standards in the United States, third-party integration platforms such as Project Meridian can provide essential flexible infrastructure.

Rapid data collection is critical to an optimized national and international response [6,32,36]. Discovery PREP addressed this need by building and piloting an electronic data capture tool that was successful in collecting coordinated and real-time multisite data to assess health system stress and evaluated treatment protocols for seasonal influenza across the United States. The lessons learned from this report should be leveraged to improve data collection efforts and provide the foundation for further investigations focused on the evolution of manual data abstraction into reliable, real-time, and automated information exchange.

## Appendix

Multimedia Appendix 1 Akido Labs Development Environment overview.

Multimedia Appendix 2 Project Meridian query status and leaderboard screenshots.

Multimedia Appendix 3 Project Meridian SMS text messaging and email communication screenshots.

## Abbreviations

CRF: case report form
Discovery PREP: Discovery Critical Care Research Network Program for Resilience and Emergency Preparedness
ICU: intensive care unit
PHE: public health emergencies
SUS: System Usability Scale
UAT: user acceptance testing

## Disclaimer

Some of the information described herein was presented in abstract form at the 48th Critical Care Congress of the Society of Critical Care Medicine, February 17-20, 2019, San Diego, California, United States, and the 49th Annual Critical Care Congress, February 16-19, 2020, Orlando, Florida, United States.
