# Supplementary figures and images for: An Electronic Data Capture Tool for Data Collection During Public Health Emergencies: Development and Usability Study
Source: JMIR Hum Factors. 2022 Jun 9;9(2):e35032. doi: 10.2196/35032 (PMC9227656; doi:10.2196/35032)

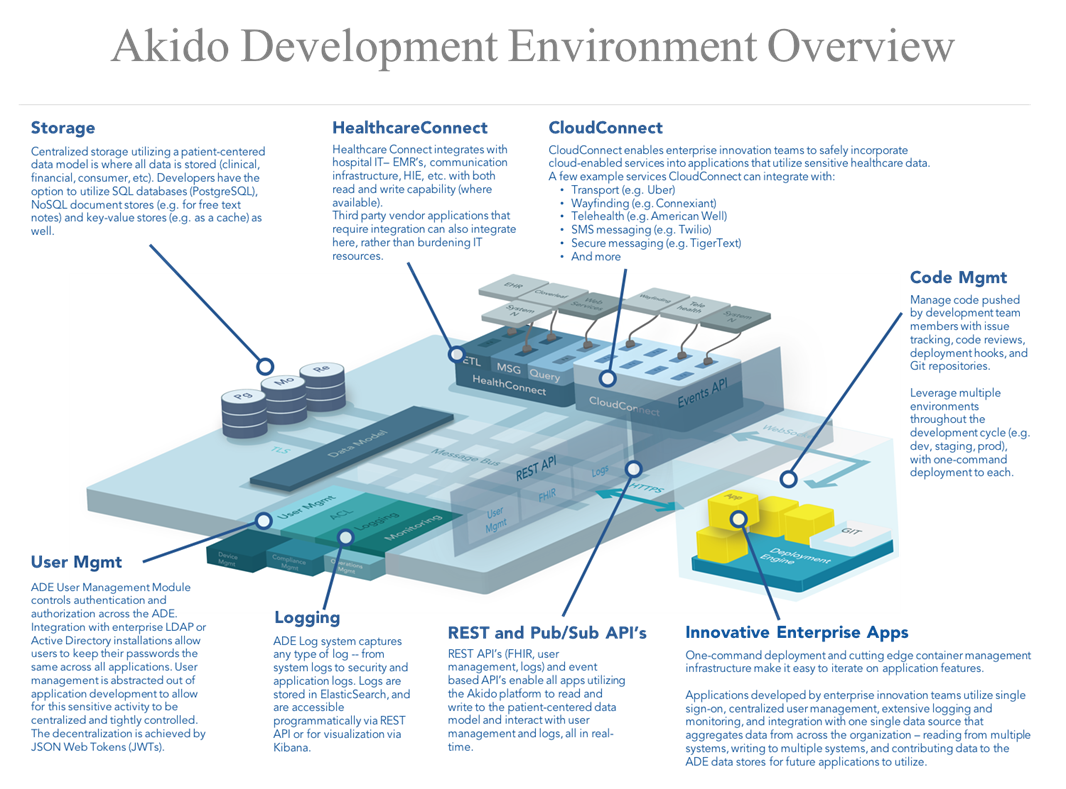

Supplement: Multimedia Appendix 1 [file humanfactors_v9i2e35032_app1.png]

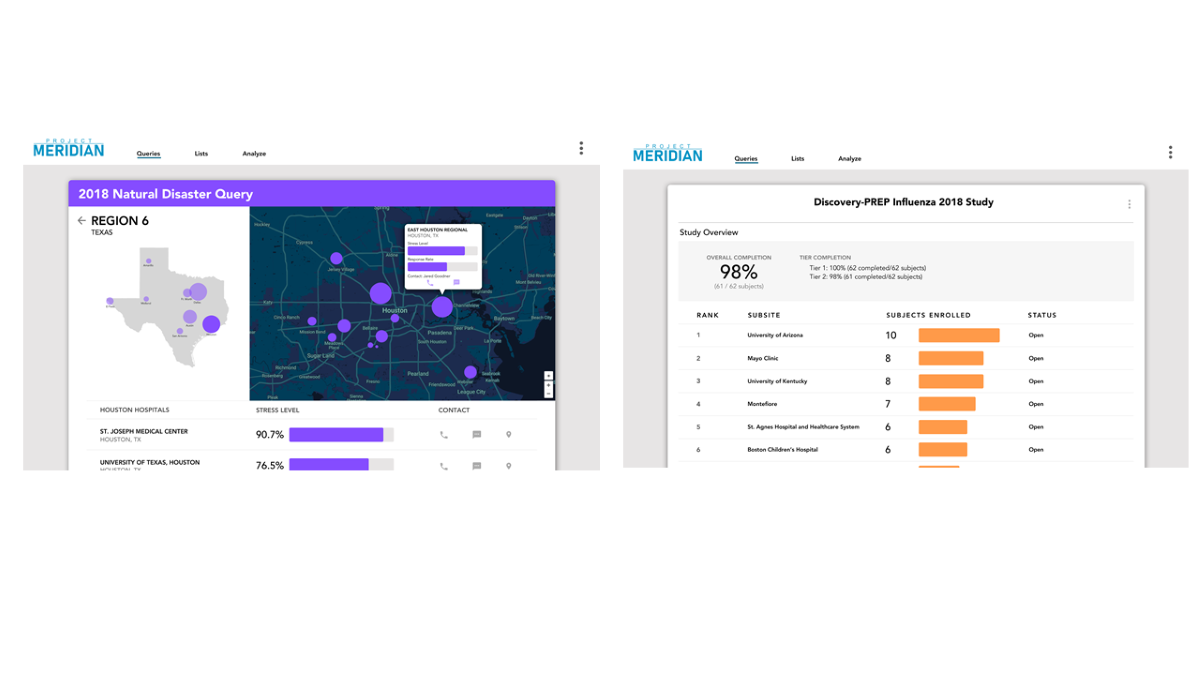

Supplement: Multimedia Appendix 2 [file humanfactors_v9i2e35032_app2.png]

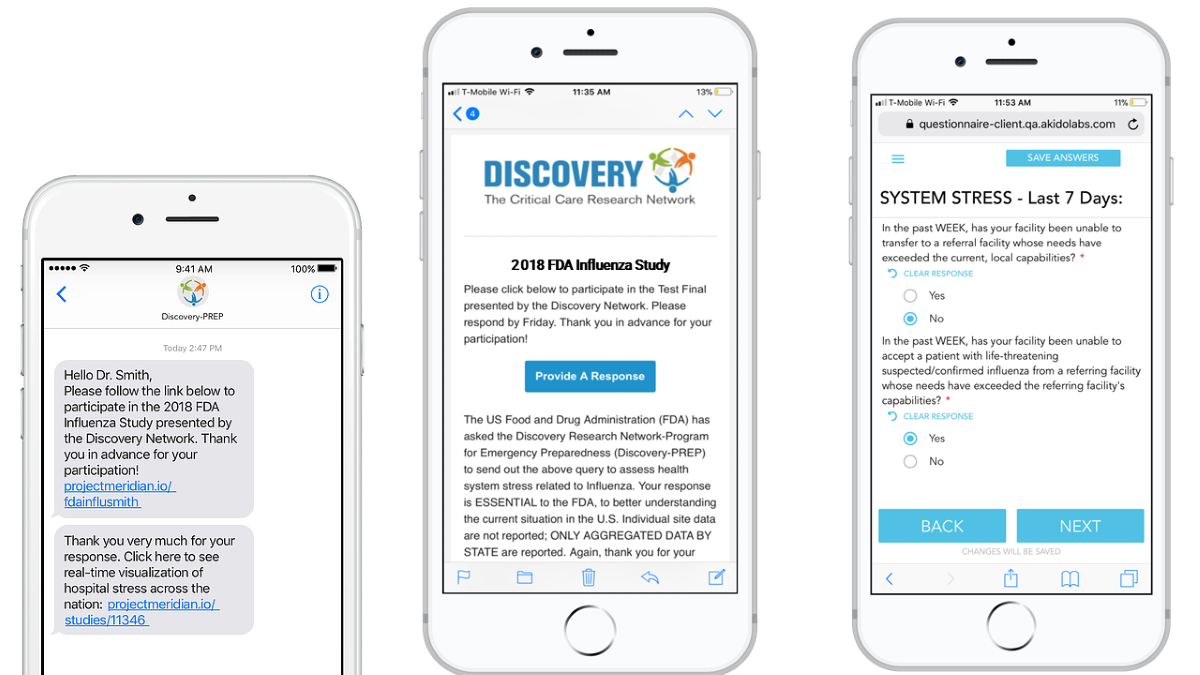

Supplement: Multimedia Appendix 3 [file humanfactors_v9i2e35032_app3.png]
